# Incident Learning and Failure-Mode-and-Effects-Analysis Guided Safety Initiatives in Radiation Medicine

**DOI:** 10.3389/fonc.2013.00305

**Published:** 2013-12-16

**Authors:** Ajay Kapur, Gina Goode, Catherine Riehl, Petrina Zuvic, Sherin Joseph, Nilda Adair, Michael Interrante, Beatrice Bloom, Lucille Lee, Rajiv Sharma, Anurag Sharma, Jeffrey Antone, Adam Riegel, Lili Vijeh, Honglai Zhang, Yijian Cao, Carol Morgenstern, Elaine Montchal, Brett Cox, Louis Potters

**Affiliations:** ^1^Department of Radiation Medicine, North Shore-LIJ Cancer Institute, Hofstra North Shore-LIJ School of Medicine, New Hyde Park, NY, USA

**Keywords:** incident learning, failure-mode-and-effects-analysis, root cause analysis, patient safety, no-fly-policy, six sigma, electronic whiteboard

## Abstract

By combining incident learning and process failure-mode-and-effects-analysis (FMEA) in a structure-process-outcome framework we have created a risk profile for our radiation medicine practice and implemented evidence-based risk-mitigation initiatives focused on patient safety. Based on reactive reviews of incidents reported in our departmental incident-reporting system and proactive FMEA, high safety-risk procedures in our paperless radiation medicine process and latent risk factors were identified. Six initiatives aimed at the mitigation of associated severity, likelihood-of-occurrence, and detectability risks were implemented. These were the standardization of care pathways and toxicity grading, pre-treatment-planning peer review, a policy to thwart delay-rushed processes, an electronic whiteboard to enhance coordination, and the use of six sigma metrics to monitor operational efficiencies. The effectiveness of these initiatives over a 3-years period was assessed using process and outcome specific metrics within the framework of the department structure. There has been a 47% increase in incident-reporting, with no increase in adverse events. Care pathways have been used with greater than 97% clinical compliance rate. The implementation of peer review prior to treatment-planning and use of the whiteboard have provided opportunities for proactive detection and correction of errors. There has been a twofold drop in the occurrence of high-risk procedural delays. Patient treatment start delays are routinely enforced on cases that would have historically been rushed. *Z*-scores for high-risk procedures have steadily improved from 1.78 to 2.35. The initiatives resulted in sustained reductions of failure-mode risks as measured by a set of evidence-based metrics over a 3-years period. These augment or incorporate many of the published recommendations for patient safety in radiation medicine by translating them to clinical practice.

## Introduction

The preparation of a treatment plan for delivery of radiation therapy to a patient requires several process steps and checks spread over about a week with interactions and handoffs between a heterogeneous set of caregivers, hardware, and software interfaces. Thus pathways for errors to propagate toward an unsafe event for any patient are multifold. Yet the rate of serious or adverse errors in radiation therapy is estimated to be around 0.2% per patient (1–4). While comparable to serious error rates in chemotherapy (5), a field that is technologically less complex than radiotherapy, the adverse error rates in radiation medicine are less favorable than those in blood transfusion and anesthesiology (3), or in aviation (6) – an industry that is cognate with radiation therapy in its hierarchical organizational structures and reliance on complex machinery. Thus while serious error rates are low, benchmarks suggest we could do better in radiation medicine.

Recommendations to do better in this regard are typically summarized in reports published by radiotherapy quality and safety organizations worldwide. A recent study of seven such reports from influential radiation therapy safety organizations worldwide yielded 117 recommendations, 61 one of which were deemed to be unique (7). Most reports with the possible exception of one were based on expert opinion rather than evidence. Twelve pertinent topics appeared in more than three of these reports. These were training, staffing, documentation, standard-operating-procedures, incident learning, communication, checklists, quality control, preventive maintenance, dosimetric audits, accreditation, minimizing interruptions, prospective risk assessments, and safety culture. While such recommendations are clearly valuable, the message to translate them uniformly, effectively, and efficiently to clinical practice in multiple departments appears nebulous as this study highlights.

There appears to be only modest improvement in error rates in medicine in general where similar broad recommendations may have been made (8, 9). In one study with 10 US based hospitals chosen on the basis of patient safety activities no significant reduction in overall rates of harm or preventable harm was reported (10), while in another, a substantial percentage of patients from tertiary care hospitals recognized for their efforts in patient safety received harm (11). On the contrary, substantial improvements have been noted where specific evidence-based approaches were used, such as with the prevention of central venous catheter bloodstream infections or reductions in mortality rates in anesthesiology (9). Focused assessments of patient safety outcomes targeted by specific evidence-based interventions and metrics may therefore be a useful complement to translate such recommendations to effective practice (9, 12).

In spite of having implemented many of these traditional recommendations, quantitative analysis of the metadata from our electronic medical records (EMR) or event-reporting database in our radiation medicine department suggested we were at a substantially higher risk than we would have perceived (13). Recognizing that not all poor processes lead to poor outcomes, and some good processes may lead to poor outcomes (14) particularly in an environment where some risk prone behavior is not discouraged, our approach was to develop a quality management (QM) framework that integrated the structure-process-outcomes approach put forth by Donabedian (15–17), knowledge of variations in clinical practice using the Deming approach (18) and event-reporting based on Codman principles (19). In order to incorporate this framework, we utilized systems engineering tools such as those used in high reliability industrial organizations and applied these to our practice (13).

In this work we reviewed incidents reported in our Aspects-of-Care (AOC) incident-reporting database to extract causes and contributory factors for known failures (20) and conducted a Failure-Mode-and-Effects-Analysis (FMEA) on our process-map (21) to predict hypothetical effects relative to patient safety. The rationale for either approach *per se* is that for every effect there must be a set of causes and for every cause there must be some set of effects (22). Based on the combined approach, we developed and implemented six initiatives for risk-mitigation with specific evidence-based metrics for evaluation that were both process and outcomes based (23, 24). We report on the sustained impact of these initiatives since these were implemented over the past few years at our facility.

## Materials and Methods

### Department structure

Our department of Radiation Medicine is spread across the New York metropolitan area over five sites within a major healthcare system. The sites that provide radiation therapy are characterized by a blend of academic, private, and community-practice traits that may be representative to some extent, of a range of practice cultures in many other departments in the country. We treat over 2000 new patients each year with external beam radiation and brachytherapy utilizing eight linear accelerators and three high-dose-rate brachytherapy systems. Our operations are paperless, driven by quality checklists (QCLs) in our EMR and policies and procedures that are updated regularly. Internal and external dosimetric audits and reviews of our programs are conducted regularly by various accreditation agencies. Our QM program encompasses all aspects of patient care using the AOC database as a catchment framework for the registry and analysis of events that represent departures from standard operations or expected patient outcomes.

### Incident-reporting and learning system (AOC database)

The AOC database is electronically available to all staff members with an anonymous reporting option. Incidents are reported with a brief narrative and associated reporting elements. The events are reviewed weekly by a multidisciplinary QM team and reported department and health-system wide on monthly and quarterly bases. The taxonomy for analysis of events evolved historically from a drop-down menu with shorthand notations for typical causes prior to 2010 to a more structured and recursive approach that mapped patient effects with underlying structure-process elements. The primary reason for the change was the observation that reviews of the incidents were being overwhelmingly attributed to just a handful of causes. A focused independent review of these however revealed that the causes typically picked were more the result of deeper issues not being considered. Thus efforts to make changes until then were not directed at the correct underlying issues, rendering our reporting system ineffective. The changes in the database taxonomy in 2010 thus allowed for more comprehensive analyses. Events are broken down into the observed patient effect (19) and the underlying structure-process factors that may have contributed to its occurrence. Patient effects include treatment delays, treatment-planning delays, treatment interruptions, patients not starting treatment, patients not completing treatments, morbidity (grade 3 or higher), mortality, preventive safety events (inclusive of near misses), and reactive safety events (inclusive of variances). The taxonomy evolved using validation studies that demonstrated improvements in the consistency of independent analysis by various members of the QM team as measured using free-marginal kappa statistics (25). These included randomly selected events that analyzers independently categorized. There have been over 2600 reports logged and analyzed since its initial use in 2007.

### Failure-mode-and-effect-analysis

Given the cascaded steps and handoffs evident in the radiation medicine process, our goal was to reorganize the chronologically arranged process-map into one that was risk-sorted for patient safety. The QCL process-map (QPM) in use at our facility and similar to others (1, 26) was cast in the framework of an FMEA spreadsheet with explicit procedural definitions. Our FMEA was based on the electronic process-map driven by the QCLs in the EMR. Thus metadata from the EMR to estimate likelihood-of-occurrence risks over any interrogated time period was potentially available.

The exercise was conducted by 15 staff members across the various functional groups in the department including radiation oncologists, physicists, dosimetrists, nurses, therapists, and one non-clinical observer. There were two to four participants in each functional group bracketing the hierarchical spread within. The participants were asked to scrutinize the process-map, prospectively consider various modes of failure for each step and list their causes and safety consequences. In addition to individual experience, the AOC database, group discussions, and other event-reporting databases or publications (27–29) served as guidelines. Events in the AOC database were considered for alternate potential manifestations regardless of reported impact. Independent responses were amalgamated after a 2-weeks response period. Risk-scoring guides were established for the three components of risk – severity, likelihood-of-occurrence, and detectability using logarithmic scales (Table 1). Similar scales have been used by others. The master spreadsheet was sent to the participants to grade each failure-mode listed in the three risk components, following clarification of the grading scheme, and collective walkthroughs of sample failure-modes. Responses were obtained within 2 weeks and discussed with the team. Average scores for each failure-mode were computed. The consistency of grades assigned by various participants was evaluated using kappa statistics. A risk priority number (RPN) was computed as the product of the three risk elements for each of the failure-modes.

**Table 1:** **Grading scheme used for FMEA exercise**.

Severity of effect		Occurrence rating	Detection
Little to no effect	None	Very low: once in 6 years or 1/18,000 quality checklists (QCLs)	Almost certain
	Very minor	Very low: once in 3 years or 1/9000 QCLs	Very high
Inconvenience	Minor	Low: once a year or 1/3000 QCLs	High
	Very low	Moderately low: once a year or 1/1500 QCLs	Moderate high
Loss or degradation of secondary function	Low	Moderate: once a quarter or 1/750 QCLs	Moderate
	Moderate	Moderately high: once a month or 1/250 QCLs	Low
Loss or degradation of primary function	High	High once a fortnight or 1/120 QCLs	Very low
	Very high	High: once a week or 1/40 QCLs	Remote
Major	Hazardous with warning	Very high: once a day or 1/15 QCLs	Very remote
	Hazardous w/o warning	Very high: more than once a day or 1/5 QCLs	Almost impossible

### Development and evaluation of risk-mitigation initiatives

Given the observed (AOC) and predicted (FMEA) propensity for either delays or defects in high-risk procedures, mitigation strategies were developed to reduce their severity, likelihood-of-occurrence, and detectability risks. The AOC findings were scrutinized to seek evidence for resident pathogens – or latent error-provoking conditions in the clinic and to ascertain if these had any bearing on the performance of the high-risk procedures (30, 31). Staffing, training, and equipment QM were deemed adequate based on external audits by accreditation agencies. Initiatives that would confer robustness in our system in regard to the pathogens regardless of violations or variability were sought (18, 30). Metrics to gage effectiveness of the initiatives, both in terms of specific outcomes and specific processes, were developed and reported to the department and health-system on a regular basis.

## Results

### Incident-reporting and learning system

Incident-reporting increased by 47% following the AOC database restructure in 2010. There was significant variation in reporting by functional group: therapists (62.9%), nurses (26.7%), dosimetrists (6.4%), physicists (1.5%), support staff (1.2%), anonymous (0.9%), and radiation oncologists (0.4%). About 89.6% of the reports were logged by therapists and nurses while 8.3% were logged by the treatment-planning team. A high level of inter-rater agreement (free-marginal multi-rater kappa score of 0.837) was noted amongst QM team members who routinely analyzed incident reports.

Patient effects reported included treatment delays (25.2%), treatment-planning delays (20.9%), incomplete treatments (14.5%), safety: preventive incidents (12.8%), treatment interruptions (6.9%), safety: reactive incidents (6.7%), mortality (5.3%), patients not starting treatment (4.2%), and morbidity (3.5%). Patient treatment-planning or initiation delays and safety incidents (preventive and reactive) comprised 65.6% of the reports in the AOC database. Under-reporting of treatment-planning delays by the treatment-planning group in the AOC database was partially compensated by reporting by the therapist group, albeit at a later day than when the delays were potentially known to be imminent to the planning team. The planning delay issues were typically identified and reported instead during the routine pre-treatment audit of plans in the EMR 2 days prior to treatment initiation conducted by therapists in our department, when expected documentation was determined to be incomplete or missing in the EMR.

Treatment-planning delays (some of which lead to treatment delays) are attributed to planning procedural delays (58%), plan verification delays (23%), environmental issues (10%), coordination-of-care with other caregivers (5%), and patient factors (4%) (Table 2). Most of the planning procedural delays resulted from the need for additional information, issues with image fusion, modifications in contours or prescriptions and coordination, and scheduling. Safety incidents are attributed to discrepancies in the EMR (33%), missing approvals (15%), patient set-up issues (12%), on-treatment check delays or defects (11%), variances (11%), missing documents (8%), patient and staff incidents related to falls, collisions (10%) (Table 3). The variance rate per patient was under 0.5% while the serious error rate was under 0.01%.

**Table 2:** **Factors contributing toward treatment-planning delays or treatment delays as reported and analyzed based on our incident-reporting and analysis system**.

Treatment-planning and treatment delays
**PLANNING PROCEDURE DELAYS (58%)**
Contour or prescription delays: additional information needed (previous RT treatment, additional images, MD peer review, new diagnostic workup or findings, pathology reviews), case complexity, late image import into TPS, management (MD availability and handoffs), re-contouring, delinquency
Plan delay: insufficient time for planning (case complexity, physics coordination, delay-rush processes), plan modifications (plan deliverability issues on Linac, prescription or constraint modifications, modality change, re-contouring), problems with plans (protocol requirements not achieved, inconsistent with directives), management (planner availability and handoffs), delinquency
Modifications to previous SIM required: fit or placement of treatment aid inadequate, changes in patient anatomy (surgical procedures, device implants), images unacceptable for treatment-planning (artifacts, anatomical coverage), patient preparation inadequate (bladder, rectal filling)
**PLAN VERIFICATION DELAYS (23%)**
Second Physics checks: fields not approved in EMR, IMRT QA delays or issues, plan documents not in EMR, problems identified with plans, physics coordination
Problems noted by therapists in V-SIM checks: problems identified with plans (ambiguous plans in EMR, dose mismatch error, incorrect DRRs, incorrect field size, delinquent patient accessory requests), plan deliverability issues, pre-treatment repeat CT required, treatment machine issues, missing approvals
**ENVIRONMENT (10%)**
Informatics issues (problems with EMR, network communications issues, PACS, TPS), scheduling, and coordination of appointments; weather related issues
**COORDINATION-OF-CARE WITH NON RT MDS (5%)**
Other procedures or MD availability: chemotherapy, admittance to hospital, blood work, dental work, erbitux therapy, heart monitoring, infection treatment, medical oncology appointments, other exams needed, neurosurgeon availability
Additional information presented: pathology reports, protocol screening, surgical consultation, tumor rounds, biopsy, laboratory tests, further diagnostic workup results
**PATIENT FACTORS LEADING TO DELAYS (4%)**
Patient not amenable to/compliant with RT procedures, receiving treatment for other health/medical problems, scheduling unsatisfactory or required change, transportation issues, personal factors, obtaining second opinions, declined MRI, debilitation, deteriorating medical conditions
**SIMULATION DELAY (1%)**
Simulation stopping events, image quality problems

*Numbers in parentheses correspond to the percentage of cases for each sub-category*.

**Table 3:** **Factors contributing toward either preventive (good catches/near misses) or reactive findings as reported and analyzed based on our incident-reporting and analysis system**.

Safety events: preventive and reactive
**DISCREPANCY IN EMR (33%)**
Dose tracking per field inconsistent with Rx, field, or DRR name mismatch, incorrect DRRs, field parameter mismatch (gantry, collimator, couch angles, fields swapped, monitor units), prescription mismatch, accessory mismatch, incorrect field size, incorrect beam energy, incorrect treatment time set-up in fields, documents for a different patient, treatment plan issues (bolus, dose computation inconsistent with prescription, images not fused, incorrect accessory factor, MLC settings, overlap with previously treated fields, plan inconsistent with directive, treatment plan for different linear accelerator plan inconsistent with simulation set-up notes), field parameters changed inadvertently on first day physics check, field size changes following system upgrade
**MISSING TREATMENT APPROVALS IN EMR (15%)**
Physician approvals (prescription, treatment plan, pathology review documentation), physics approvals (IMRT QA documentation, treatment fields, second check approval of treatment plan, planner approval of treatment plan), multiple approvals missing, second physics check approval completed prior to field parameter entry completion, incomplete quality checklist tasks
**PATIENT SET-UP (12%)**
Port film issues, machine clearance issues, insufficient field coverage, difficulties with treatment aids/devices, incorrect treatment device fabrication, missing devices, Vac-loc bag deflation, incorrect localization methodology, set-up difficulties due to changes in patient anatomy or preparation, patients could not tolerate set-up
**ON-TREATMENT CHECKS DELAYED/MISSING/INCORRECT (11%)**
Patient *in vivo* (nanodot) measurements not documented in EMR, first day physics check delayed, not documented or documented prior to first day treatment, weekly physics chart check note missing, quality checklists (e.g., laterality) not completed
**VARIANCE (INCORRECT DOSE DELIVERED, INCORRECT VOLUME IRRADIATED OR BOTH) (11%)**
Incorrect treatment field used (handoff), not all treatment fields delivered (fields hidden), incorrect shifts applied (handoff), bolus not used, incorrect gantry angle used (override, wide tolerance tables, communication between EMR and Linac communications), incorrect fractionation delivered (treatment calendar), incorrect block used (text overlay on DRR), incorrect monitor units (incorrect use of MU calculation sheet, missing physics check, tray factor), incorrect collimator angles, partial treatment delivered (machine limitations, EMR and Linac communications), incorrect energy used, incorrect couch angle, incorrect accessory used, incorrect field size (inadvertent asymmetric to symmetric setting change on first day physics check), IGRT localization data for different patient used, incorrect fiducial markers used, pacemaker patient received one treatment without rhythm strip, patient simulated without physician documentation in EMR, incorrect SSD (override), field block by couch top
**MISSING/INCORRECT DOCUMENTS IN EMR (8%)**
Pathology report missing prior to V-SIM, pacemaker alert and/or dosimetry information missing, IMRT QA report missing, treatment plan missing, insurance authorization documents missing, patient identification documents missing, consultation documents missing, second physics check document missing
**PATIENT INCIDENT (7%)**
Fall/slippage/collision, rapid response or emergency procedures unrelated to radiation therapy (breathing, O2 saturation drop, blood pressure), injury (procedural complications – applicator insertion or removal, removal of ekg lead, removal of HDR unit prior to removing catheter), coordination-of-care (pre-operative radiation delivered for subsequently delayed surgery)
**STAFF INCIDENT (3%)**
Fall/slippage/collision; injury while assisting patient, exposure to bodily fluids or matter, electrical shock (field engineer)

### Failure-mode-and-effect-analysis

Based on the Landis-Koch criteria (32), Kappa-scores for severity-risks (0.533) and detectability risks (0.629) were fair or good for the highest risk-processes but poor for the likelihood-of-occurrence risk (0.140). The highest RPN based on this exercise was 127 with all participant responses considered and 151 when responses for specific process steps were limited to those who routinely performed them.

**Figure 1:**
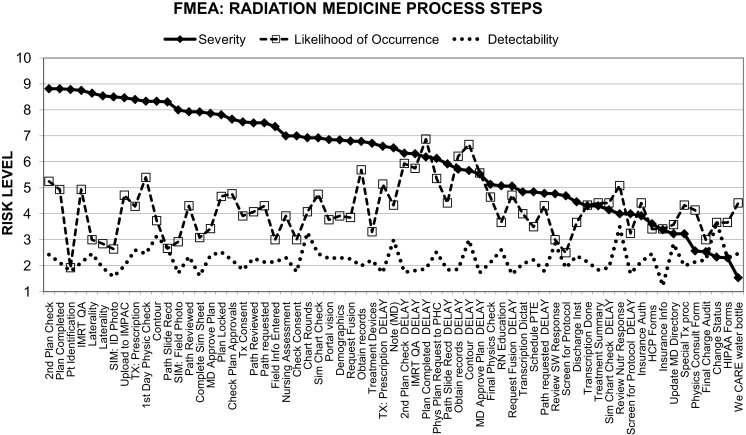
**Decomposition of our process-map in radiation medicine into three components of risk levels based on prospective failure-mode analysis**.

**Figure 2:**
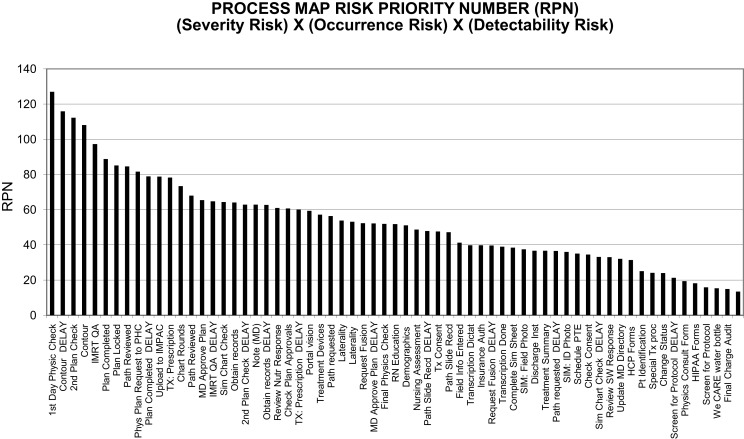
**Composite risk probabilities of failure-modes from combined components of risk elements**. Based on inputs from all staff members.

The procedures with the highest severity-risks and RPNs included contours, prescription, treatment plan completion, and transfer to the EMR, MD plan approvals, IMRT QA, second physics checks, pathology review, patient consent, laterality, and first day physics checks (Figures 1 and 2).

### Baseline risk profile

**Figure 3:**
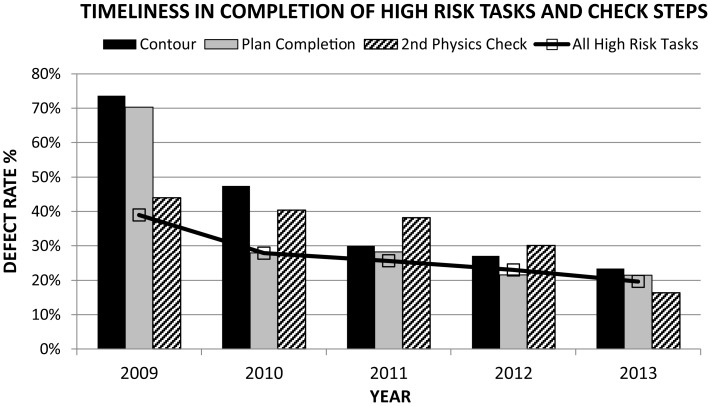
**Mitigation of the likelihood-of-occurrence risks for high-risk-process steps over time based on actual data extracted from the EMR**.

**Figure 4:**
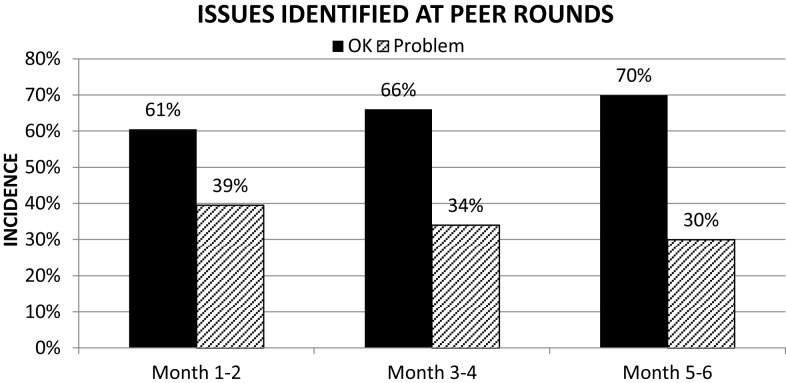
**Mitigation of defects increased process control for high-risk-process steps (contours and prescription in care pathways) over time**.

**Figure 5:**
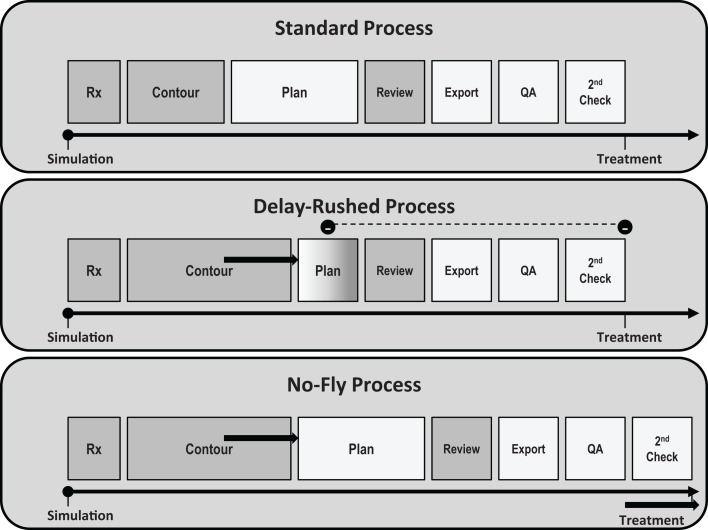
**Delay-rushed processes**. On the upper panel is a qualitative plot of a nominal timeline for task completions – the width of each block corresponding to the time needed for each task. Not changing the expected treatment date, in the event of upstream task delays, would require shortening the time for completion of the downstream tasks (middle panel) and thus exacerbated time pressures that could lead to errors. On the other hand, extending the treatment date proactively to account for the upstream task delay would restore the downstream timelines (bottom panel) and a “treat safely” first culture.

A baseline review of metadata extracted from our EMR indicated that approximately 40% of all the high-risk procedures identified on the FMEA were completed with delays (Figure 3). In particular, 70% of contours and plan completion procedures were delayed. Approximately 39% of all cases presented at pre-treatment-planning peer review rounds presented with issues that required contour completion, modification, or directive changes (Figure 4). Latent risk factors that contributed to these included communication, procedural compliance, and time pressures (Tables 2 and 3). The latent risk factors created an environment where upstream high-risk procedures were delayed, but the overarching tendency to treat on time necessitated a hastened completion of downstream high-risk tasks. The net effect of the latent risk factors was the prevalence of a local delay-rushed culture (Figure 5) incompatible with patient safety goals.

**Figure 6:**
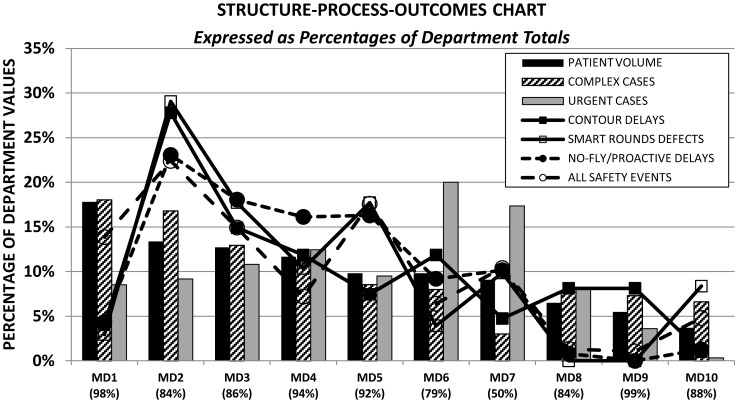
**Variability amongst radiation oncologists in process execution and patients effects in the framework of department caseload distributions**. All percentages are quoted based on department totals for each category. Numbers quoted near the MD designations correspond to the fraction of patients seen by each MD at their prominent practice site amongst the four sites of the department.

Substantial variability in execution of high-risk procedures was noted amongst staff as illustrated by the structure-process-outcomes chart (Figure 6). Patient treatment volume was chosen as an indicator for structure (17, 33, 34). Three of the 10 radiation oncologists who collectively carried 36% of the department patient volume were associated with 50% of the department contour delays and 65% of the issues (contour modification or inconsistent directives) identified at pre-treatment-planning peer reviews, 52% of all reports logged in the AOC database, 56% of treatment delays, 55% of all safety incidents, and 46% of department variances; thus more than their volume share would predict. In contrast one radiation oncologist who carried 18% of the department volume had 4% of the department contour delays, 3% of the peer review issues, 12% of all AOC reports, 9% of all treatment delays, 14% of all safety incidents, and 18% of department variances; well below or at par with the treatment volume share. For the remaining radiation oncologists process delays or peer review issues and AOC effects were at par with or less than total department shares of patient volumes.

### Risk-mitigation strategies and their impact

Analysis of the AOC database, FMEA, and baseline risk profile resulted in six risk-mitigation initiatives to minimize process delays, defects, and variability amongst staff. These were standardization of care pathways (35) and toxicity grading (36) to reduce the severity risk; pre-treatment-planning peer review rounds (37), and a no-fly-policy (38) to reduce the likelihood-of-occurrence and detectability risks; an electronic whiteboard (39) as a process mapping and error detection tool to enhance communications, reduce detectability risks, and augment the AOC database; and the use of six sigma metrics to gage operational efficiencies in the context of timely completions and reduced variability (13).

The respective metrics used to gage effectiveness were compliance-with-directive usage, kappa-scores for inter-rater reliability of toxicity grading schemes, fraction of cases presented at pre-treatment-planning peer review rounds with no issues, proactive and no-fly treatment delay rates, incident-reporting rates and high-risk-process task delay rates, or *Z*-scores.

**Figure 7:**
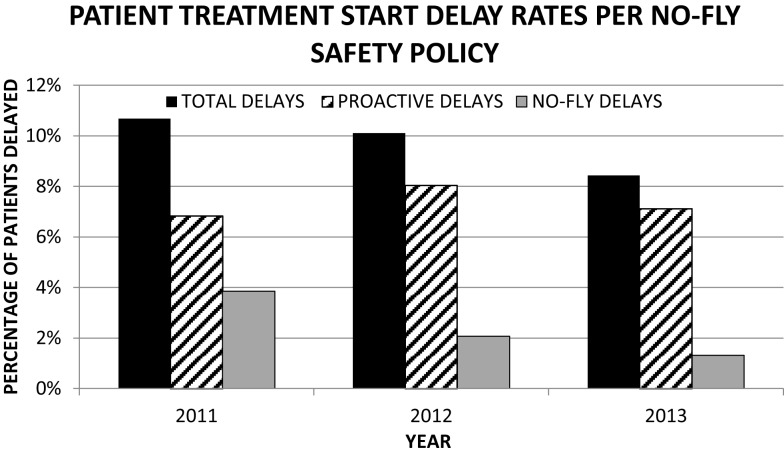
**Time trends of treatment delays since the introduction of our no-fly-policy**.

There has been a sustained rate of improvement across all metrics evaluated over the past 3 years. The compliance with the use of care pathways has steadily increased to 97%. Inter-rater reliability for toxicity grading has improved by a factor of two using kappa statistics. Defect rates in timely completion of high-risk tasks have dropped steadily from 39 to 20% (Figure 3). The percentage of cases presented at pre-treatment-planning peer review rounds with no defects identified has increased from 61 to 70% in the first 6 months of its implementation (Figure 4). Current defect detection rates for contours and care pathways are approximately 7 and 9% respectively of patients presented at rounds. Proactive and no-fly delay rates have dropped from 12 to 8% of all new patient starts (Figure 7). Incident-reporting in the whiteboard by the treatment-planning team has increased more than three times in 9 months of usage relative to 6 years of usage of the AOC database with no increase in the observation of adverse events. Operational *Z*-scores have improved from 1.78 to 2.35.

## Discussion

Moving toward safer radiotherapy practice requires an active surveillance of associated failures, their causes and effects, and evidence-based approaches to mitigate them. Surveillance may be conducted reactively, such as via the analysis of incidents reported at any given institution, in multi-institutional databases or published reports (1, 27–29, 40–54). Alternatively, it may be conducted proactively using probabilistic safety assessment tools, risk matrices, or failure-mode-and-effect analysis (29, 55–60). Understanding the nature, cause and effects of errors in radiation medicine is confounded by the involvement of human or cultural factors and their interactions with systems (30, 61), varying human perceptions during incident-reporting and analysis (62), under-reporting, or skewed reporting of incidents amongst functional groups (3), and practical limitations of anticipating all possible trajectories for failure propagation in complex systems (22). This is partially illustrated in one recent study, where it was shown that a large fraction (42%) of incidents reported were not predicted by the FMEA conducted at the same institution (3) and is likely to be true for other studies including ours.

From a practical perspective, what neither reactive nor proactive surveillance approaches can do independently is reveal all underlying causes of specific effects observed or all possible adverse effects of specific causes (22). Much may be learned then from the combined surveillance approach. Using probabilistic safety assessment tools on 443 failure-modes, a multidisciplinary task group of the Ibero American FORO of Nuclear and radiation safety agencies have shown that 90% of potentially catastrophic accidental exposures involving multiple patients could be attributed to as few as eight event sequences with misunderstanding of delineated treatment volumes, initial treatment sessions, patient positioning, treatment delivery, and treatment-planning being the most vulnerable steps in the process (29, 56). Continued incident-reporting on a national or international level may reveal a similar cluster of risk-heightened sequences and therefore direct mitigation strategies more effectively.

A limitation that affects both forms of surveillance is that of the differences in perception by staff members. The same incident report could be interpreted differently by different staff members analyzing the report. This in turn has important consequences on incident learning or relative risk assessment and therefore the legitimacy or effectiveness of resulting corrective actions. The difference in perception was exemplified when the kappa-scores for consistency in analysis of a set of test cases from our AOC database amongst members of the QM team (typically at 0.837) dropped with the addition of a new team member (to 0.747), and by the low kappa score (0.140) for the likelihood-of-occurrence risks for the high-severity risk tasks during the FMEA exercise. This emphasized the need for the continual assessment of inter-rater reliability in the use and development of taxonomies for incident learning. In regard to the FMEA, there was a wider variation in perceptual differences for the likelihood-of-occurrence assessments for two reasons. First, the FMEA was conducted by members across all functional groups and thus some of the failure-modes for a particular type of procedure were less understood by or familiar to those who did not routinely perform them. As errors started by one professional group however are often caught by another (63), an observation exemplified several times in our AOC database, the group perspective was an important step in determining and assessing various modes of procedural failures. Second, many of the failure-modes identified were hypothetical in nature, and thus the estimation of their likelihood-of-occurrence with existing controls in place was subject to individual perception bias. To reduce the effects of these limitations, RPN scores were also calculated by restricting the likelihood-of-occurrence risks to those who routinely performed the procedures and by finding ways to measure actual defect rates by querying the EMR directly where possible. The scales used for FMEA are ordinal scales and are subject therefore to rater perception. The RPN computation is thus somewhat flawed as it involves the multiplication of grades based on ordinal data. Hence our assessment of the risk profile for the various process steps during the FMEA was based on collective consideration of the severity-risks and the semi-quantitative RPN scores rather than on the latter alone.

Despite the limitations of failure analyses mentioned above, the use of various incident-reporting systems, differing taxonomies, and FMEA study designs (including grading schemes used) at different institutions, in comparing our findings (Figures 1 and 2; Tables 2 and 3) with those of others referenced above, we have noted several similarities for the types of incidents observed, the causal relationships and the process steps where high-risk sub-steps appear to be clustered. Incident learning and FMEA studies at our institution have shown us that failures occur due to delays in the completion of the radiation medicine process steps or defects relative to standard-operating-procedures in key steps along the way and that the nature of process execution is dependent on the structural set-up of the department and coordination with other caregivers or the patients themselves. There were three main contributors to failures including treatment delays and safety findings in our database.

The first was the timeliness and accuracy of the execution of high-risk-process steps identified in our FMEA. In particular, contours, prescription, the completion of the treatment plan and second checks are complex in nature, requiring many inter-related subtasks, pre-requisite information, and handoffs, and have a tight coupling with observed treatment delays or safety incidents (Tables 2 and 3) (31, 64). Delays and defects in completing these tasks are attributed not just to staff delinquencies but to a large extent also the need for obtaining the requisite information at the right time from the right source. Structural barriers to this include inadequate procedural training or skills, ineffective handoffs or communications, improper scheduling or coordination of staff and equipment failures. For approximately 40% of the variances noted in our system, the errors were determined to have germinated at various stages of the treatment-planning preparation, clustered around these tasks. Thus despite multiple defenses-in-depth serving as quality control checkpoints (plan reviews, second physics checks, verification simulation, timeouts, and initial treatment checks), quality was not fully assured and errors were allowed to propagate. Second, our review of the structure-process-outcomes metrics highlights variability in practice amongst our radiation oncologists (Figure 6). A similar trend was noted for planners and physicists. As with the limited cluster of high-risk-process steps, the propensity toward risk is elevated for a handful of the staff where high-risk task delays and defects as well as patient effects noted in the AOC database were exacerbated relative to others despite no significant differences in their share of patient volumes, case complexity or urgency. Finally, cultural tendencies to prioritize time pressures for treatment initiation elevate the risk profile by creating local traps and recurrent patterns for delay-rushed practices and thus error-provoking conditions. Prior to 2010 75% of treatment-planning delays in the high-risk steps did not culminate in treatment delays – this dropped by a factor of two in the transition phase leading to the initiation of the no-fly-policy. Multiple incidents were reported with incomplete high–risk procedures while the patient was awaiting treatment initiation, suggesting the site culture somehow supported delay-rushed practices instead of focusing on proactively addressing underlying systemic deficiencies. Thus high-risk task delays or defects, staff variability and delay-rushed tendencies were the three main error-provoking factors in our study.

Risk-mitigation was therefore geared toward these three factors, using the mitigation strategies of the FMEA process – namely reduction of associated severity metrics, likelihood-of-occurrence and the likelihood of detection of errors if these occurred. The main focus was on building robustness within our radiation medicine process with the awareness that it may be formidable to completely eliminate failures (65) but that we may be better oriented to contain their damaging effects by injecting quality control and assurance measures between consultation and completion of planning stages and enhancing defenses-in- depth (66).

Mitigation of the severity risk was approached by enhancing standardization of care pathways and the grading of adverse effects. Non-compliance with treatment protocols and variability in practice has been shown to lead to inferior outcomes (67, 68). To avoid *ad hoc* care, we instituted detailed evidence-based treatment pathways that encompassed all domains of care including prescription, simulation, planning, nursing interventions and follow-up assessments (35). These were based on the Institute of Medicine, and quantitative analysis of normal tissue effects in the clinic (QUANTEC) guidelines (4). In order to ensure that patients would be evaluated for toxicities in a standardized way regardless of caregiver (69, 70), we incorporated the Common Terminology Criteria for Adverse Effects (CTCAE V4.0) grading scheme from the National Cancer Institute into our EMR (36, 71). These initiatives were aimed toward reducing defects in the completion of high-risk tasks due to care based on individual caregiver experience rather than published evidence. Incorporating the standards into the EMR provided us with a baseline reference with which to compare pathways chosen for specific patients and thus a means to detect and assess differences and reduce variability in practice.

To reduce the likelihood-of-occurrence and detection risks, three initiatives were introduced. Pre-treatment-planning peer reviews were instituted on a daily basis with all faculty, treatment-planning and scheduling team members to review patient charts prior to the initiation of treatment-planning (37). Similar rounds at other institutions demonstrate the value added by peer review (72). The goal was to detect errors in the care pathways as well as contours delineated in the treatment-planning systems for specific patients. The standardized care pathways were based on all faculty inputs and thus provided a framework for managing potentially contentious discussions during rounds as well as exploring opportunities for improvement based on new published evidence in the literature. The rounds served as a checkpoint for the initiation of treatment-planning so that planning would commence after reconciliation of identified defects.

Coordination-of-care amongst caregivers was enhanced by the creation of an electronic whiteboard that was used for peer rounds and for efficiently coordinating all treatment-planning tasks for all patients at all sites of the department in a more transparent manner than what was available in the EMR (39). Traditionally staff members were accustomed to mainly reviewing QCL items assigned just to them in the EMR. This reduced simultaneous awareness of gaps in the planning process by others. With the whiteboard, alerts for potential delays in all high-risk planning tasks for specific patients were made more obvious to all members of the planning team without the need for additional mouse clicks.

Given the paucity of reports in the AOC database by members of the planning team, the whiteboard additionally served as a platform to report errors identified on peer rounds as each case was reviewed. A substantial increase in incident-reporting resulted from the use of the whiteboard as an augment to the AOC database. This does not reflect an increase necessarily in the incidence of events, rather the increase in the reporting of these events. It has been reported that departments that register more events in such systems actually have fewer events that lead to patient harm (3, 73). Three factors contributed toward this improvement. First, the electronic whiteboard was used as an operational tool to navigate daily pre-treatment-planning rounds where all staff members involved in the plans of all patients being reviewed on a certain day were present. In addition to navigating rounds, its immediate access during rounds provided an efficient interface to summarize the review for each patient and also to register issues. Second, the reporting interface within the whiteboard was simpler to use as it did not require onerous and duplicitous data entry such as patient demographic information or associated data, since this data was already available. Thus the overhead in reporting was substantially reduced compared to the AOC database. This enhanced reporting efficiency not just during rounds, but at all times. For planning staff members, selecting a drop-down entry in an already open electronic whiteboard was less of a barrier to reporting than the need to separately open the AOC database and complete all entries. Third, any event identified during pre-treatment-planning rounds was required to be logged with all planning staff present. This requirement served as a forcing function for staff members to complete event entries.

Defect rates as well as delay rates of all procedures were gaged using *Z*-scores – a standard practice in high reliability industries (13). These metrics were computed previously with metadata from the EMR but are now routinely computed with data from the whiteboard. *Z*-scores for all high-risk tasks were routinely reported to all staff members at monthly QM meetings using control charts so as to increase awareness of underlying issues and highlight individual staff operational performance improvement needs. The *Z*-score combines the accuracy with which tasks are completed based on specification limits as well as the variability in task completion. The use of the *Z*-score has provided a means of evaluating our progress in the same framework used by high reliability industries that strive toward six sigma levels of operations.

Finally to mitigate risks associated with rushed completions of high-risk tasks, we implemented a “No-fly-policy” with stopping rules that proactively delayed patient treatment starts or prevented treatment starts in the event such delay-rushed processes would have occurred (38). Overall, there has been a steady trend showing a drop in high-risk task delays as well as a drop in patient treatment initiation delays. The policy provides a robust mechanism to mitigate potentially unsafe treatment starts regardless of variability in high-risk task completions or staff performance. It has transformed the culture from “treat on time” first to “treat safely” first.

An oft quoted goal in radiation medicine is to deliver the right dose to the right target while minimizing dose to organs at risk. Much has been done by way of algorithm or technique development toward meeting that goal, but little in terms of addressing comprehensive structure-process-outcome deficiencies. Given our baseline risk profile (likely similar to that for other departments), we felt we needed more than a set of policies or checklists in order to transform our culture to one that was actively focused on safety. Driving these risk-mitigation initiatives has challenged traditional norms of operations such as expediting treatment initiation in delay-rushed environments or sustaining care pathways that are more experience based than evidence-based. Therefore their implementation has met with substantial cultural barriers for adoption. Working practices evolve over decades, and changing them with such initiatives creates uncertainty. The inertia of sustaining past cultures and arguments for not changing tend to perseverate (74). Direct persuasion only goes so far (75). In anticipation of these barriers, our initiatives were not implemented abruptly, but via transitional phases focused on staff feedback, education, training, and communication (76). The goal was to introduce them in a manner that would support the staff to embrace the measures by the time of formal implementation. Regardless of the barriers, our focus on patient safety, combined with statistical process control, regular event database reviews and staff meetings, and use of quantitative metrics that has been instrumental in realizing these changes and crossing barriers. We have seen sustained improvements over the past 3 years of implementation in our department. Institution of the following items has helped drive our department toward improvements in patient safety: (i) standardization of treatment care pathways, (ii) standardization of toxicity grading, (iii) institution of prospective pre-treatment-planning peer reviews, (iv) process management for treatment-planning components, (v) assessment of operational efficiencies, and (vi) enforcement of the No-Fly-Policy. We believe that most centers can augment their safety programs by complying with and instituting some or all of these initiatives. By doing so, they can use the work from this study to build a culture of safety without necessarily replicating the more time-consuming aspects of the study. Yet, for others, there is value in validating our results.

## Conflict of Interest Statement

The authors declare that the research was conducted in the absence of any commercial or financial relationships that could be construed as a potential conflict of interest.
